# Mitochondrial DNA D‐loop sequence analysis reveals high variation and multiple maternal origins of indigenous Tanzanian goat populations

**DOI:** 10.1002/ece3.8265

**Published:** 2021-11-01

**Authors:** Athumani Nguluma, Martina Kyallo, Getinet Mekuriaw Tarekegn, Rose Loina, Zabron Nziku, Sebastian Chenyambuga, Roger Pelle

**Affiliations:** ^1^ Tanzania Livestock Research Institute (TALIRI) Dodoma Tanzania; ^2^ Sokoine University of Agriculture Morogoro Tanzania; ^3^ Biosciences Eastern and Central Africa‐International Livestock Research Institute (BecA‐ILRI) Hub Nairobi Kenya; ^4^ Department of Animal Breeding and Genetics Swedish University of Agricultural Sciences (SLU) Uppsala Sweden; ^5^ Department of Animal Production and Technology Bahir Dar University Bahir Dar Ethiopia

**Keywords:** demographic history, genetic variation, haplogroups, indigenous goats

## Abstract

The Small East African (SEA) goat are widely distributed in different agro‐ecological zones of Tanzania. We report the genetic diversity, maternal origin, and phylogenetic relationship among the 12 Tanzanian indigenous goat populations, namely Fipa, Songwe, Tanga, Pwani, Iringa, Newala, Lindi, Gogo, Pare, Maasai, Sukuma, and Ujiji, based on the mitochondrial DNA (mtDNA) D‐loop. High haplotype (*H*_d_ = 0.9619–0.9945) and nucleotide (*π* = 0.0120–0.0162) diversities were observed from a total of 389 haplotypes. The majority of the haplotypes (*n* = 334) belonged to Haplogroup A which was consistent with the global scenario on the genetic pattern of maternal origin of all goat breeds in the world. Haplogroup G comprised of 45 haplotypes drawn from all populations except the Ujiji goat population while Haplogroup B with 10 haplotypes was dominated by Ujiji goats (41%). Tanzanian goats shared four haplotypes with the Kenyan goats and two with goats from South Africa, Namibia, and Mozambique. There was no sharing of haplotypes observed between individuals from Tanzanian goat populations with individuals from North or West Africa. The indigenous goats in Tanzania have high genetic diversity defined by 389 haplotypes and multiple maternal origins of haplogroup A, B, and G. There is a lot of intermixing and high genetic variation within populations which represent an abundant resource for selective breeding in the different agro‐ecological regions of the country.

## Graphical abstract

The indigenous goats in Tanzania belong to Small East African (SEA) goat breed widely distributed in different agro‐ecological zones. This study examines their origin, phylogenetic relationship among the different populations and the demographic history. The goats have high maternal genetic diversity defined by 389 haplotypes and multiple maternal origins of haplogroups A, B, and G. There is a lot of intermixing and high genetic variation within populations which represent an abundant resource for selective breeding in the different agro‐ecological regions of the country.

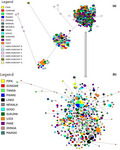

## 1 BACKGROUND

The Tanzanian goat (*Capra hircus*) population is currently estimated to be 24.1 million (NBS, 2020) with 97% comprising of indigenous goats belonging to the Small East African (SEA) goat breed (MLF, 2017). Due to their adaptability to different climatic conditions, the indigenous goats are widely distributed in almost all agro‐ecological zones of Tanzania. Goats are important species for the livelihood of the rural farming communities especially those residing in arid and semi‐arid areas of Tanzania where other agricultural activities are not feasible. They are raised mainly for meat and manure, and as a source of income (Chenyambuga et al., 2012). Additionally, goats play different socio‐cultural and traditional roles as gifts, dowry payments, and spiritual offerings. Despite their wide distribution in Tanzania, the indigenous goats have low productivity in terms of growth and milk production. Efforts to improve their productivity have mainly focused on crossbreeding with exotic germplasm which has proved to be unsustainable in the long run. Selective breeding utilizing the indigenous adapted animals would have a sustainable impact on the productivity of the animals (Syrstad & Ruane, 1998). This requires the animals to be characterized to understand the level of genetic diversity and the relationship between different animal populations (FAO, 1992; FAO/UNEP, 1998). The limited information on the characteristics of indigenous goats in Tanzania is mostly based on the phenotypic features which can be subjective and dependent on the environment which makes it difficult to distinguish between populations (Falconer & Mackay, 1996). Previous efforts to study genetic diversity of indigenous goats in Tanzania have focused on only a few populations from few agro‐ecological zones using microsatellite markers (Chenyambuga et al., 2012; Nguluma et al., 2018), making it difficult to draw conclusion on population structure of the indigenous goats of Tanzania in relation to agro‐ecological zones of the country. Studying the genetic history of domestic animals can provide crucial clues about past events and main pathways used for commercial transport of the animals in historical times and therefore provide us with information about their genetic structure and relationship within and among populations. Information from such studies is needed in designing and implementing conservation and improvement programs for indigenous goats. This study, therefore, was designed to determine the genetic diversity, maternal origin, and phylogenetic relationship of 12 populations of the indigenous goats in Tanzania using the mitochondrial DNA (mtDNA) D‐loop region.

## 2 MATERIALS AND METHODS

### 2.1 Sample collection and DNA extraction

**FIGURE 1:**
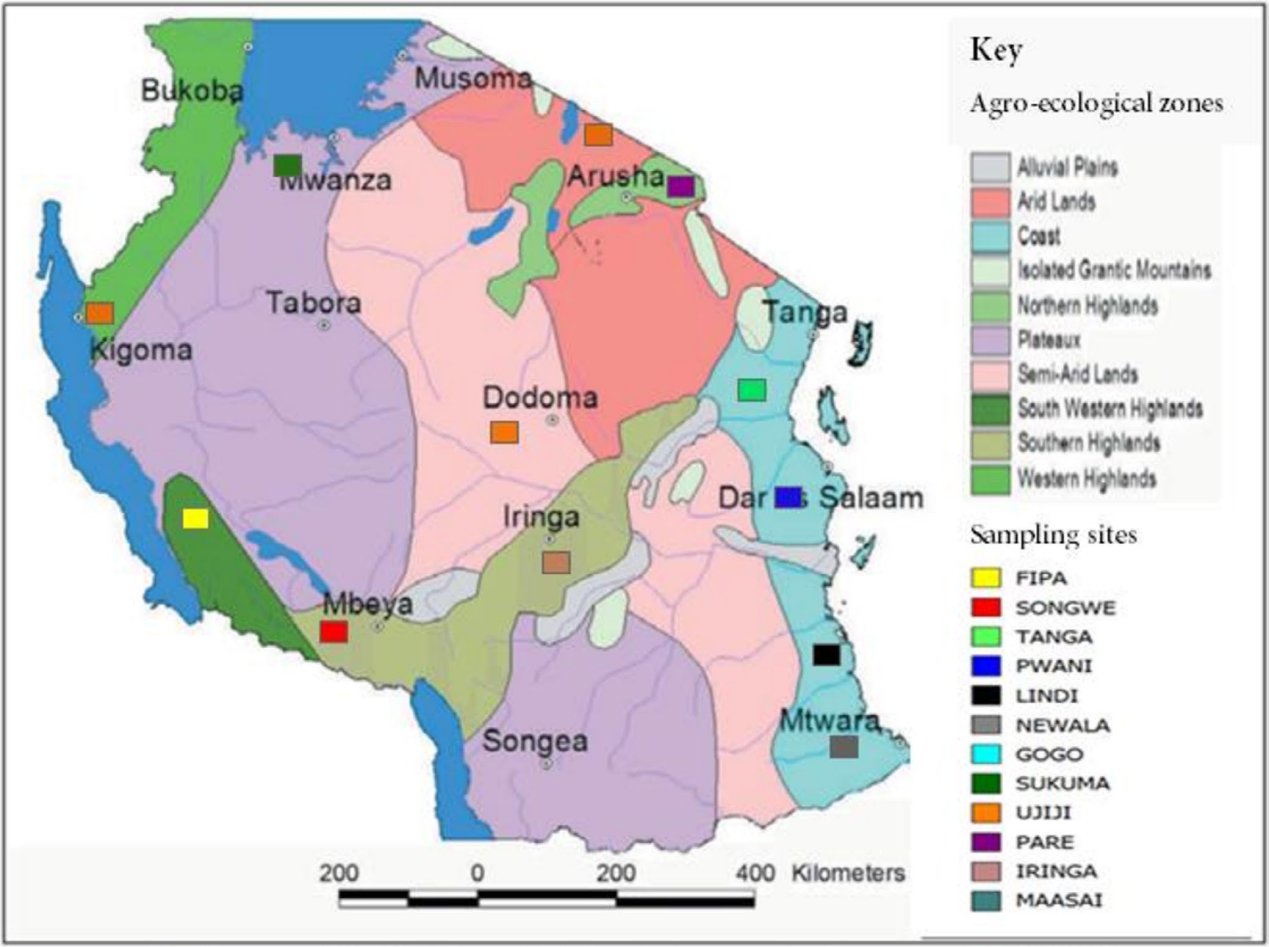
Map showing populations sampled from the different agro‐ecological zones of Tanzania. Map adapted from Sokoine University of Agriculture (2014)

A total of 627 blood samples were collected from unrelated indigenous female goats from major geographical agro‐ecological zones of Tanzania (Figure 1) representing 12 populations. The goat populations were Fipa (*n* = 44) from Southwestern highlands, Songwe (*n* = 34) and Iringa (*n* = 35) from Southern highlands, Tanga (*n* = 33), Pwani (*n* = 40), Newala (*n* = 49), and Lindi (*n* = 46) from Coast, Gogo (*n* = 73) from semi‐arid, Pare (*n* = 67) from northern highlands, Maasai (*n* = 72) from arid, Sukuma (*n* = 67) from plateaux, and Ujiji (*n* = 67) from western highlands zone. Total genomic DNA was isolated from blood using the QIAGEN DNeasy Blood & Tissue Kit and TANBead OptiPure Blood DNA Extraction Kit (Taiwan Advanced Nanotech Inc.) according to the manufacturer's protocol. The concentration and purity of extracted DNA were assessed using the Nanodrop1000 spectrophotometer (Thermo Fisher Scientific).

### 2.2 PCR amplification and sequencing

The primer pair 6807‐F 5′‐ACCAGAAAAGGAGAATAGCC‐3′ and 8173‐R 5′‐GGTACACTCATCTAGGCATT‐3′ flanking the mtDNA D‐loop region were designed in this study to amplify the complete D‐loop region. PCR amplification was performed in a total volume of 15 μl containing Phusion High‐Fidelity PCR Master Mix (Thermo Fisher Scientific Inc.), 0.1 pM of each primer, 2% DMSO (Applied Biosystems), and 50 ng of template DNA. Amplification was carried out in a GeneAmp PCR System 9700 thermal cycler using the following cycling conditions: initial denaturation at 98ºC for 30 s, followed by 35 cycles at 98ºC for 10 s, 62ºC for 30 s, and 72°C for 1 min, with a final extension of 72°C for 10 min. The PCR products were fragment separated on a 1.5% agarose gel pre‐stained with 0.25× GelRed (Biotium) and visualized under UV light. The PCR fragment sizes were estimated using O’Gene 100‐bp DNA ladder (Thermo Fisher Scientific Inc.). The ExoSAP‐IT™ PCR Product Cleanup Reagent (Thermo Fisher Scientific Inc.) was used to purify PCR products before Sanger sequencing.

### 2.3 Data analysis

Sequences were manually edited and aligned using Clustal W program (Larkin et al., 2007) in CLC Workbench 8.0.3 (CLC Bio‐Qiagen). The *Capra hircus* D‐loop mtDNA sequence (GenBank accession number GU223571) was used for reference mapping during sequence assembly. Haplotypes were determined with DnaSP v5 (Librado & Rozas, 2009), and basic diversity parameters were computed for each population using Arlequin 3.5 (Excoffier & Lischer, 2010). Twenty‐two goat mtDNA reference sequences belonging to six known haplogroups/lineages (Naderi et al., 2007) were downloaded from GenBank and used for haplogroup identification. Sequences of 201 individuals belonging to 29 goat populations from nine other African countries (Table 1) were also downloaded from the GenBank and were included in the analysis. The phylogenetic relationship between individuals and populations was assessed based on the Tamura–Nei distance model (Tamura & Nei, 1993) using the neighbor‐joining (NJ) algorithm implemented in MEGA6 (Tamura et al., 2013). The assessment was done using all the generated sequences, the 22 reference sequences and sequences of wild goat ancestors (*capra aegagrus*, *Capra caucasica*, *Capra cylindricornis*, *Capra falconeri*, and *Capra sibirica*) with the bootstrap percentage computed after 1000 replications. To confirm the maternal origin and relationship between the Tanzania goat populations and populations from other African regions, median‐joining (Bandelt et al., 1999) network was drawn using Network V10.2.0.0 (www.fluxus‐engineering.com). The reference sequences representing the six haplogroups are determined based on the variation in the first hypervariable region of the d‐loop which is 481 bp. Therefore, sequences generated in the Tanzanian goats were first truncated to 481 bp in order to accommodate the reference sequences then used in the construction of the NJ tree and MJ network. To determine population genetic structure within and among populations, analysis of molecular variance (AMOVA) was performed using Arlequin version 3.0 (Excoffier & Lischer, 2010). Likewise AMOVA was performed to determine the amount of genetic variation between the different regions of the African continent. The African regions considered were East Africa (Kenya), North Eastern Africa (Ethiopia), West Africa (Nigeria), North Africa (Egypt, Algeria), Sothern Africa (South Africa, Namibia, Zimbabwe, Mozambique).

**TABLE 1:** Geographical location and characteristics of other African goat populations included in the study

Country	GenBank accession number	Reference
Kenya	KP120622–KP120681	Kibegwa et al. (2015)
Ethiopia	KY747687–KY747691; KY747989–KY747993	Tarekegn et al. (2018)
Mozambique	AJ317804–AJ317809; EF618240–EF618241	Luikart et al. (2001), Naderi et al. (2007)
Zimbabwe	AJ317802–AJ317803; EF618545–EF618546	Luikart et al. (2001), Naderi et al. (2007)
Namibia	EF618242–EF618245	Naderi et al. (2007)
Nigeria	KJ466206–KJ466236, KJ466237–KJ466262	Awotunde et al. (2015)
Egypt	AJ317780–AJ317783; AJ317795–AJ317801; EF617711–EF617728; EF618220	Luikart et al. (2001), Naderi et al. (2007)
Algeria	AJ317777–AJ317779	Luikart et al. (2001)
South Africa	AJ317812–AJ317815; AJ317819–AJ317820; AJ317844; AJ317821–AJ317822; EF618351–EF618356; KJ466263–KJ466273	Luikart et al. (2001), Naderi et al. (2007), Awotunde et al. (2015)

History and demographic dynamics were investigated through mismatch distribution patterns (Rogers & Harpending, 1992) complemented by Fu's Fs (Fu, 1997) and Tajima's *D* (Tajima, 1989) statistics calculated using the infinite sites model in Arlequin v3.5.

## 3 RESULTS

### 3.1 mtDNA D‐loop variation and genetic diversity

The complete goat mtDNA D‐loop region analyzed in this study corresponds to nucleotide positions 15431 to 16643 of the *C*. *hircus* reference sequence (GenBank accession number GU295658.1). From the 627 sequences generated for the 12 Tanzanian goat populations, 276 polymorphic sites were identified of which 223 were substitutions (214 transitions, 9 transversions) and 69 were indels. The polymorphic sites defined 389 haplotypes in total and of these 308 were unique whereas 81 were shared between individuals from at least two different populations. Maternal genetic diversity parameters for Tanzanian goat populations are presented in Table 2. All populations showed high genetic diversity indicated by the haplotype diversity ranging between 0.9485 ± 0.011 in Newala to 0.9945 ± 0.001 in Sukuma goat population while haplotype proportion (number of haplotypes in relation to the sample size) was in the range of 59.2% in Newala to 95% in Fipa populations. Nucleotide diversity was the largest for Songwe (0.0162 ± 0.037) and lowest for Lindi (0.0120 ± 0.031) goat population. Twenty‐six individuals had sequences with ambiguous nucleotides at various positions, and Ujiji goats had the highest number of individuals with ambiguous nucleotides (*n* = 14). The ambiguous nucleotides were mainly found at the beginning and toward the end of the sequence reads which was lowly variable. Analysis was done with and without the sequences with ambiguity, and it was observed that they did not produce different results and therefore the sequences were retained during analysis.

**TABLE 2:** Maternal genetic diversity of 12 Tanzanian indigenous goat populations from the analysis of the HV‐I region of the mtDNA *d*‐loop

Population	*N*	*H*	*H*_d_ ± SD	*π* ± SD	Haplogroups and number of individuals (%)		
					A	B	G
Fipa	44	35	0.9749 ± 0.006	0.0145 ± 0.051	36 (81.8)	0	8 (18.2)
Songwe	34	29	0.9763 ± 0.005	0.0162 ± 0.037	25 (73.5)	1 (2.9)	8 (23.5)
Tanga	33	28	0.9734 ± 0.006	0.0144 ± 0.033	25 (75.8)	2 (6.0)	6 (18.2)
Pwani	40	38	0.9848 ± 0.003	0.0151 ± 0.035	32 (80)	2 (5)	6 (15)
Iringa	35	31	0.9789 ± 0.005	0.0169 ± 0.061	26 (74.3)	3 (8.6)	6 (17.1)
Maasai	71	67	0.9909 ± 0.002	0.0130 ± 0.044	60 (84.5)	0	11 (15.5)
Newala	49	29	0.9485 ± 0.011	0.0128 ± 0.033	43 (87.8)	0	6 (12.2)
Lindi	46	29	0.9565 ± 0.009	0.0120 ± 0.031	41 (89.1)	2 (4.3)	3 (6.5)
Gogo	73	65	0.9874 ± 0.002	0.0139 ± 0.047	60 (82.2)	1 (1.4)	12 (16.4)
Sukuma	67	51	0.9945 ± 0.001	0.0134 ± 0.046	58 (86.6)	2 (3.0)	7 (10.4)
Pare	67	54	0.9847 ± 0.003	0.0139 ± 0.126	57 (85.1)	0	10 (14.9)
Ujiji	68	53	0.9619 ± 0.007	0.0135 ± 0.044	59 (86.8)	9 (13.2)	0
Overall	627	389	0.9945 ± 0.001	0.0139 ± 0.046	522 (83.3)	22 (3.5)	83 (13.2)

Abbreviations: *H*, number of haplotypes; *H*_d_, haplotype diversity; *N*, sample size; SD, standard deviation; *π*, nucleotide diversity.

### 3.2 Phylogenetic relationship

**FIGURE 2:**
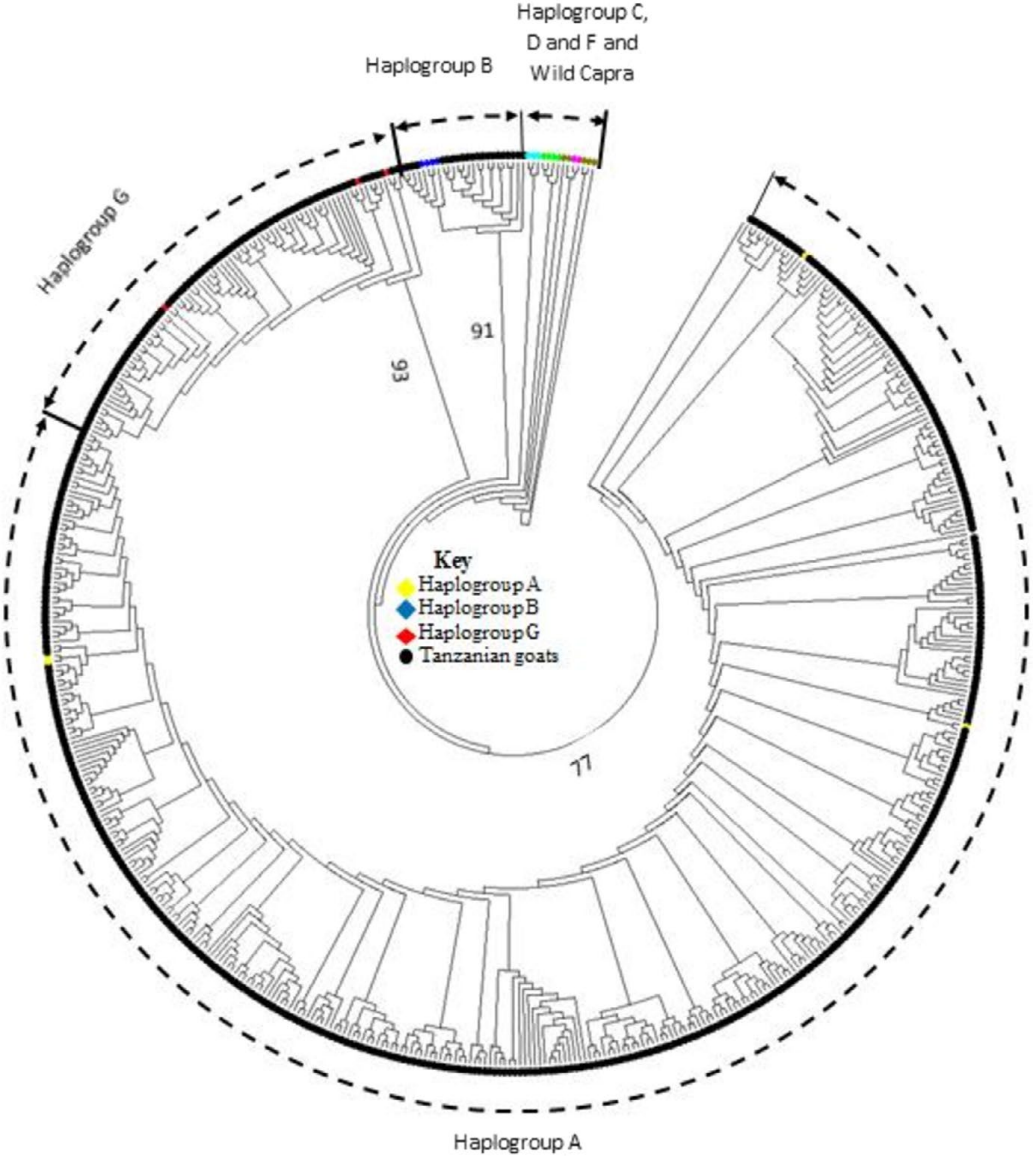
Neighbor‐joining tree constructed using the HV1 region of the mtDNA D‐loop of 12 Tanzanian goat populations, reference sequences representing six haplogroups observed in goats and five wild ancestors. Bootstrap values below 75% are not shown

**FIGURE 3:**
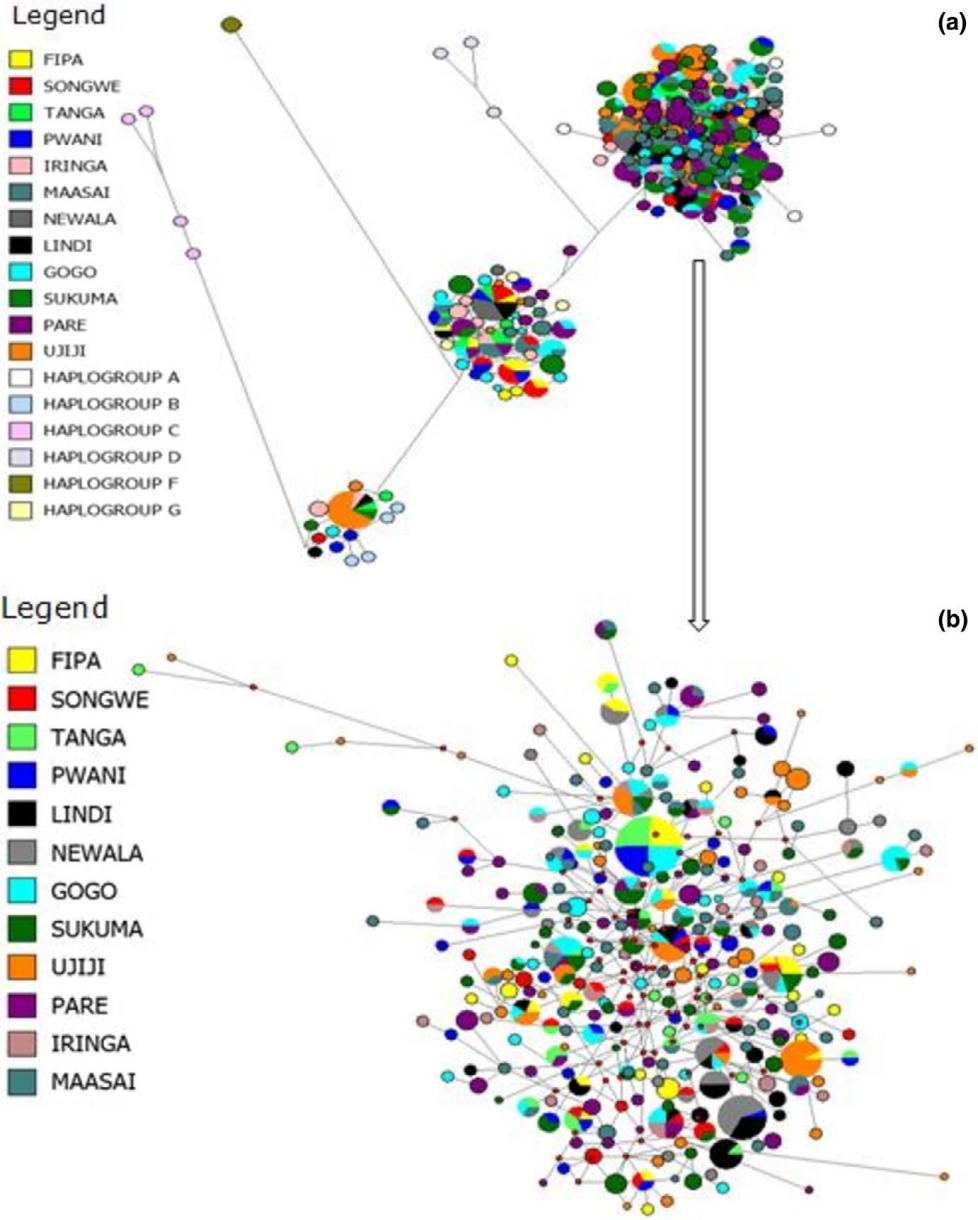
Median‐joining network analysis for indigenous goat populations of Tanzania. (a) Network topology of six goat haplogroups, and (b) predominant haplogroup A showing sharing of haplotypes between different goat populations. The area of the circle is proportional to haplotype frequency

Phylogenetic analysis was conducted to assess the relationship among the 12 Tanzanian goat populations and assign individual goats to their respective maternal origins. The hypervariable region I (HV1) of the mtDNA D‐loop which is 481 bp long corresponding to the positions 15,737 to 16,189 on the *C*. *hircus* complete mitochondrial reference sequence (accession number GU295658.1) was used to construct the neighbor‐joining (NJ) tree and median‐joining (MJ) network. Both, the NJ tree (Figure 2) and MJ network (Figure 3) showed that the Tanzanian goat populations were classified into three distinct groups which represented Haplogroup A, B, and G. Haplogroup A was the most predominant and contained 334 haplotypes representing 522 individuals drawn from all goat populations. Haplogroup G contained 83 individuals from all populations except Ujiji and was comprised of 45 haplotypes. Haplogroup B had only 22 individuals and 10 haplotypes representing 3.5% of all goats mostly from the Ujiji population. An MJ network used to assess the relationship among the Tanzanian goat populations revealed that Maasai and Gogo populations had the highest number of shared haplotypes (*n* = 10) while only one haplotype was shared by Ujiji‐Newala, Ujiji‐Pwani, Sukuma–Lindi, and Sukuma–Tanga pairs of populations. The most commonly shared haplotype (H119) was shared among six populations namely Newala, Gogo, Iringa, Ujiji, Maasai, and Sukuma while the most frequent haplotype (H85) occurred in 14 individuals from Newala, Pwani, and Lindi populations.

**FIGURE 4:**
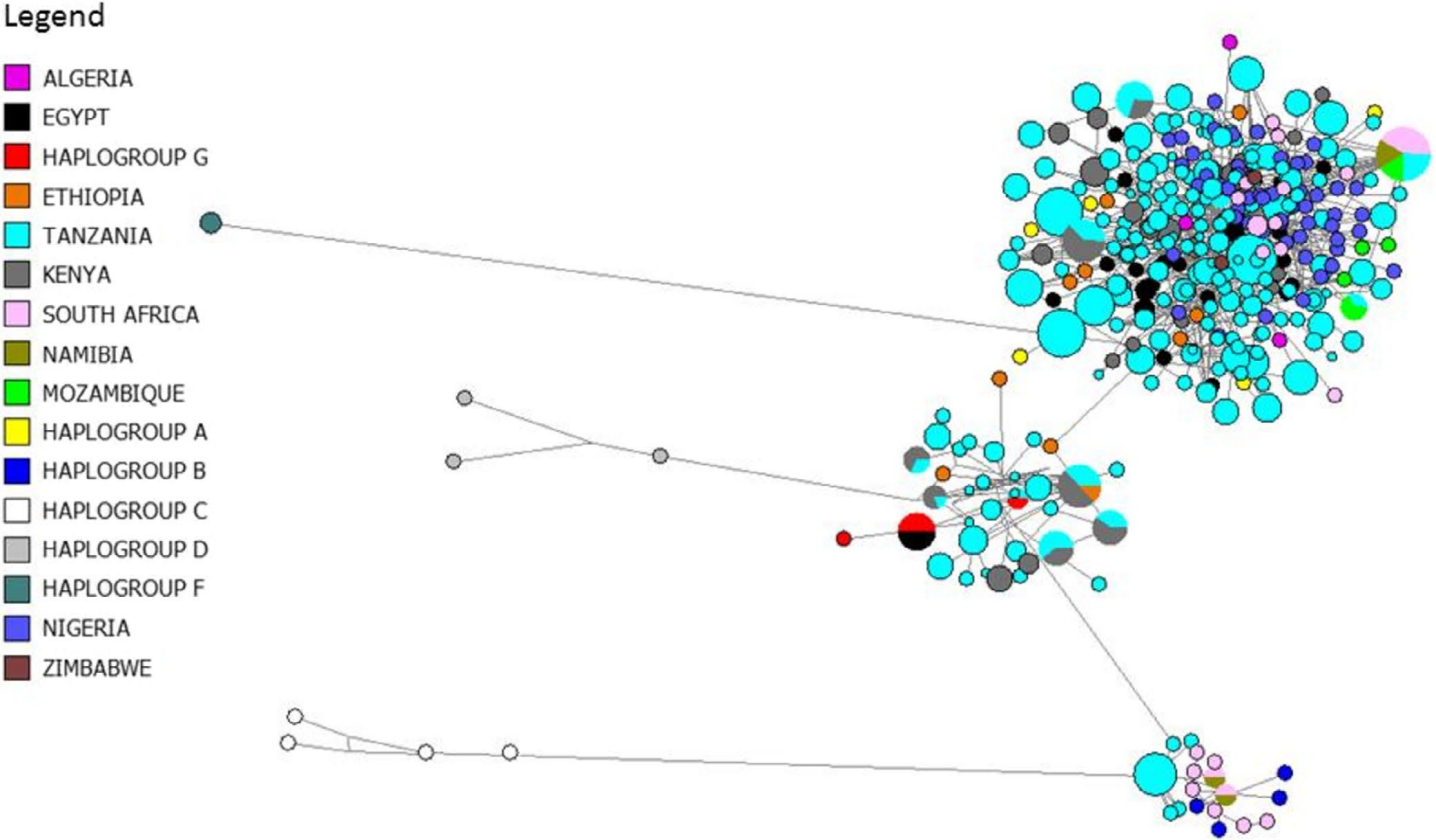
Median‐joining network based on the haplotypes of HV1 control region of indigenous Tanzanian goats and goats from nine different African countries. The different colors are related to the geographical origin, and the area of the circle is proportional to haplotype frequency

To provide a wider resolution of the phylogenetic relationship between the Tanzanian goat populations with those of other regions of the African continent, an MJ network was constructed using sequences from Tanzanian goats in this study and goat populations from across nine African countries retrieved from the GenBank database (Figure 4). Three clusters were again formed with global haplogroup A comprising of individuals from all countries. Haplogroup B was comprised of individuals from Tanzanian, South African, and Namibian goat populations. Haplogroup G was occupied by individuals from Tanzania, Egypt, Ethiopia, and Kenya. The MJ network further revealed that Tanzanian goats shared many haplotypes with Kenyan goats and a few with goats from South Africa, Namibia, and Mozambique. There was no sharing of haplotypes observed between individuals from Tanzanian goat populations with individuals from North and West Africa (Nigeria, Egypt, and Algeria).

### 3.3 Population structure

The AMOVA results shown in Table 3 below revealed that a non‐significant (*p *> .05) proportion (2.8%) of the total genetic variation occurred among the goat populations while a significant proportion (97.2%) of the total genetic variation was observed within the Tanzanian goat populations. On the continental level, a significant genetic variation was found within the regions (12%) and among the regions (87.57%).

**TABLE 3:** Results of AMOVA based on the analysis of the complete mtDNA d‐loop in 12 Tanzanian goat populations

Source of variation	Tanzanian goat populations		African regions’ goat populations^a^	
	Among populations	Within populations	Among regions	Within regions
df	11	621	4	327
Variance	0.259	8.91	0.84407	5.94381
% of Variation	2.83	97.17	12.43	87.57
*p* Value	.063	.0028*	.048*	.019*

Abbreviation: df, degrees of freedom.

*Statistically significant (*p* < .05)

^a^ Four geographic regions defined as Tanzania, East Africa, North Africa, West Africa, and South Africa.

### 3.4 Population demographic history

**FIGURE 5:**
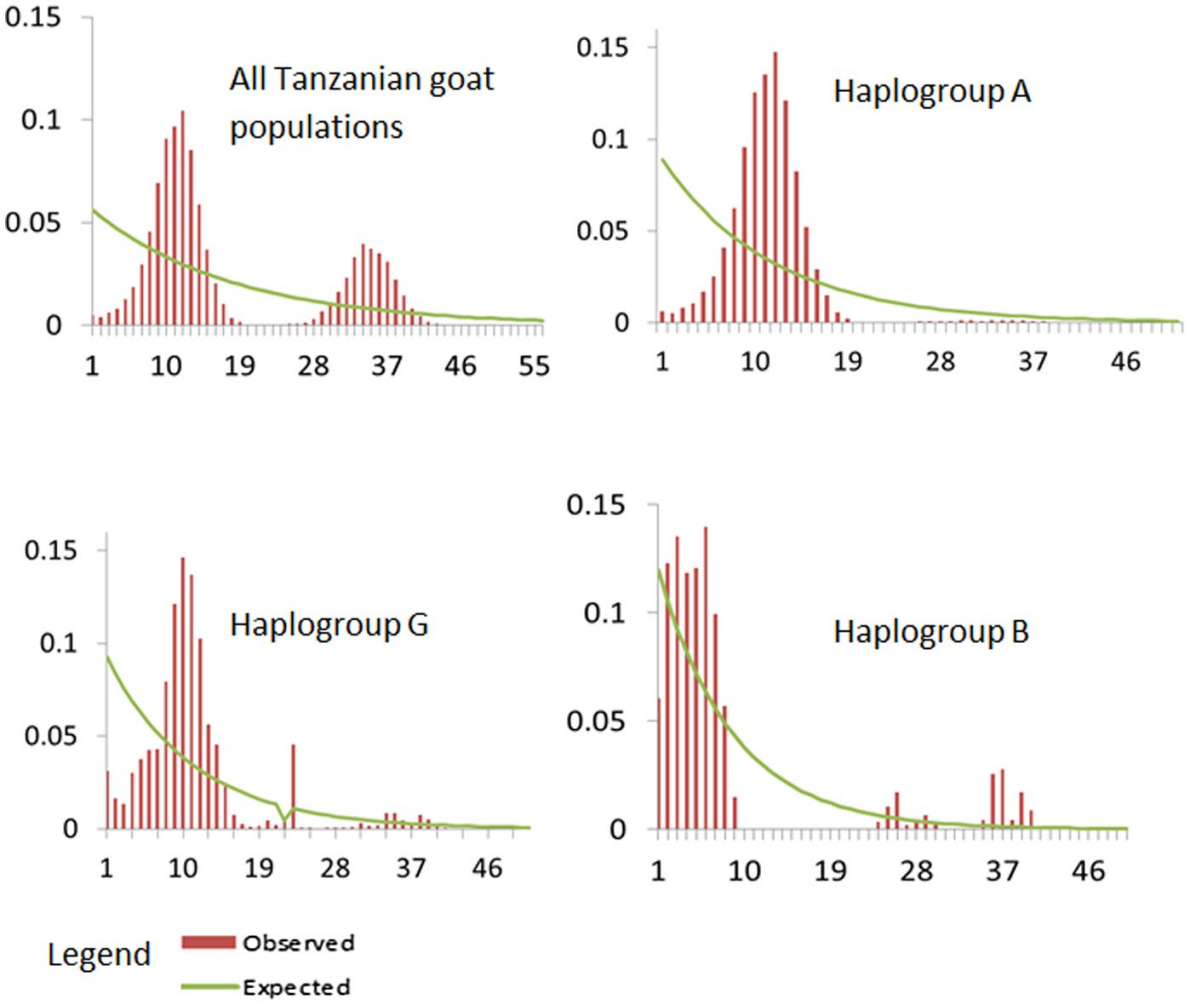
Mismatch distribution graphs for the 12 Tanzanian indigenous goat populations and Haplogroups A, B, and G analyzed in this study. The *x*‐axis shows the number of pairwise differences, and *y*‐axis shows the frequency of the pairwise comparisons

Past population expansion events were inferred based on the patterns of the mismatch distributions (Figure 5) and neutrality test estimates presented in Table 4. For each population and haplogroup, the mismatch distributions showed ragged and bimodal patterns and the observed pattern did not deviate significantly from that expected under a null hypothesis model of either spatial or demographic expansion except for Haplogroup A which had a smooth and unimodal pattern. The Tajima's *D* values for the goat populations were negative and not significantly different from zero for all populations while Fu's Fs estimates were not statistically significant except for Gogo and Maasai whose Fu's Fs values were negative and significant. Furthermore, the SSD and Happending's raggedness index (*r*) computed to ascertain the goodness of fit of the mismatch distributions varied among populations (Table 4). Estimates for SSD and raggedness index values were positive and non‐significant for all populations. Similarly for all haplogroups, the SSD and raggedness index were positive and non‐significant while the Tajima's *D* values were all negative and non‐significant.

**TABLE 4:** Population demographic parameters estimated from the analysis of the complete mtDNA D‐loop in 12 Tanzanian goat populations

Population	*N*	SSD	Raggedness index (*r*)	Tajima's *D*	Fu's Fs
Fipa	44	0.021	0.010	−0.132	−0.778
Songwe	34	0.026	0.013	−0.167	0.814
Tanga	33	0.033	0.014	−0.134	0.300
Pwani	40	0.017	0.006	−0.484	−2.461
Iringa	35	0.070	0.012	−0.6	−0.80
Maasai	68	0.011	0.005	−0.771	−18.972 (0.004)
Newala	49	0.023	0.011	−0.253	2.184
Lindi	46	0.011	0.014	−0.635	1.458
Gogo	67	0.015	0.005	−0.648	−14.543 (0.022)
Sukuma	73	0.011	0.006	−0.627	−5.644
Pare	72	0.008	0.004	−0.547	−6.788
Ujiji	67	0.017	0.009	−0.126	−8.623
Haplogroup A	522	0.018	0.008	−1.364	−24.06(0.004)
Haplogroup B	22	0.016	0.008	−1.589	−1.044
Haplogroup G	83	0.019	0.007	−1.165	−14.069 (0.00)

Abbreviations: *N*, sample sizes; SSD, sum of squared deviations.

## 4 DISCUSSION

### 4.1 Genetic diversity and maternal origin

This study is the first assessment of the genetic diversity and population structure that considers goat populations from all agro‐ecological zones of Tanzania where indigenous goats are raised and provide the first insight into the genetic history of these goat populations. Previous studies on genetic diversity using microsatellite markers (Chenyambuga et al., 2002; Nguluma et al., 2018) only considered few populations representing few agro‐ecological zones. All goat population studied showed high diversity as depicted by high within‐population haplotype and nucleotide diversity. Other studies reported haplotype diversity ranging from 0.95 to 1 for other African goat populations (Awotunde et al., 2015; Kibegwa et al., 2015; Kivila et al., 2018). The high genetic diversity observed in this study may partly be attributed to reasons mentioned previously including high mutation rate of the control region, multiple maternal wild ancestors (Naderi et al., 2007), and capture of a large part of the wild diversity during domestication (Benjelloun et al., 2011). The overall ratio of transitions: transversions (28.7:1) revealed a heavy transition bias even higher than the 17:1 and 16.7 ratios reported before in domestic goats (Joshi et al., 2004; Luikart et al., 2001).

Using the available goat mtDNA haplogroup classification system (Naderi et al., 2007), three haplogroups (A, B, and G) were found among the indigenous Tanzanian goat populations. Predominance of haplogroup A in Tanzanian goat populations was expected as it is the most diverse and ancient (Naderi et al., 2007), and its wide distribution was consistent with the world scenario described in previous studies (Joshi et al., 2004; Liu et al., 2009; Sultana et al., 2003) in all goat breeds in the world. Haplogroup B was observed in low frequency in the Tanzanian goat populations except in Maasai, Pare, and Fipa populations in which it was completely absent. Haplogroup B is mostly found throughout Central and South Asia where it likely originated from (Chen et al., 2005; Colli et al., 2015; Naderi et al., 2007) and was distributed to other locations through human migration or commercial trade. Haplogroup G which was detected in East and Northeast Asia is the second most predominant type considering number of individuals and frequency distribution among the Tanzanian goat populations. Similar to our observations, Al‐Araimi et al. (2017) found rare presence of haplogroup B while Haplogroup G was the second most predominant after Haplogroup A among the Arab peninsula goat populations. Haplogroups C, D, and F have been reported in Europe (Naderi et al., 2007), and the absence of these haplogroups in the analyzed samples may indicate that the indigenous goats of Tanzania do not share a recent common ancestor with the European goat breeds and that there is no or little introgression of European germplasm into Tanzanian indigenous goats. This was expected given that mtDNA is maternally inherited and the few crossbreeding programs involving European dairy goat breeds in Tanzania use breeding bucks or semen. Additionally, such programs are implemented in areas with intensive goat production systems in which only dairy goats are raised in small numbers.

Results obtained in this study further support the concept of multiple maternal origins of domestic goats (Joshi et al., 2004; Luikart et al., 2001). Classification of the Tanzanian indigenous goats into three distinct haplogroups could be interpreted as evidence that they come from three separate genetically distinct maternal populations, or from one origin which is an extremely large population containing three highly divergent maternal lineages. According to Hassan (2000), Gifford‐Gonzalez and Hanotte (2011), domestic goats were first introduced into the African continent through the Mediterranean coast, Red Sea Hills, overland via the Sinai Peninsula and Nile Delta. The arrival of mtDNA haplogroups to Tanzania might have occurred through two types of routes: continental routes via the north gateway of the Arabian Peninsula and maritime routes via the Indian Ocean. Egypt is reported to be one of the historical entry points of domesticated animals into the African continent; therefore, the introduction of goats into Tanzania might have been through southward movement from Egypt through Sudan, Ethiopia, and Kenya. Similar observations were made by Tarekegn et al. (2018) and Al‐Araimi et al. (2017) when tracing the route of dispersal of goats into Ethiopia and Oman, respectively, from the origin of domestication which is considered to be southwest Asia (Payne & Wilson, 1999). Evidence for the north gateway route is the presence of Haplogroup G in Egypt, Kenya, and Ethiopia and sharing of some haplotypes between Tanzanian and Kenyan goats.

The arrival of goats into Tanzania via a marime diffusion route through the Indian Ocean from Asia is supported by the presence of Haplogroup B in Tanzanian goats and from South Africa, Namibia, Mozambique, and its absence in the Ethiopian and Kenyan goat populations. Furthermore, sharing of haplotypes between the Tanzanian goat populations and goat populations from Southern African countries implies that they have the same maternal origin and came via the same route.

There was no specific distribution pattern of goat populations in the different agro‐ecological zones in haplogroup A since each population was represented in the haplogroup. Haplogroup B on the other hand was dominated by Ujiji goats while Newala, Fipa, Pare, and Maasai goats were completely absent. Haplogroup G had members from all populations except the Ujiji goat population. It can be reasonably concluded that the indigenous goats in Tanzania came from different maternal populations and later mixed when spreading to different parts of the country.

### 4.2 Population structure and phylogenetic relationship

An AMOVA carried out for Tanzania indigenous goat mtDNA revealed a comparative lack of genetic structure, supporting a geographic distribution trends observed in previous studies of mtDNA diversity in goats (Chen et al., 2005; Joshi et al., 2004; Luikart et al., 2001; Naderi et al., 2007, 2008; Sultana et al., 2003; Tarekegn et al., 2018), in worldwide dataset, the Indian subcontinent, China, and Africa. The AMOVA results revealed high variation within the Tanzanian goat populations but very low and insignificant among population variation. The AMOVA results are further supported by a median‐joining network, in which the haplotype distribution pattern did not cluster according to population or agro‐ecological zones. These findings are somehow consistent with previous observations by Nguluma et al. (2018) who, based on microsatellite analysis, observed variation among four Tanzanian goat population to be insignificant though slightly higher (8%) than in this study. The low genetic variation observed between Tanzanian goat populations could be attributed to intermixing of animals across geographical regions due to pastoralism (Mwambene et al., 2014; Tenga et al., 2008), trade between people of different regions, and cultural influences like dowry payments. High within‐population variation observed in the present study could be contributed by uncontrolled mating and lack of selective breeding practiced by smallholder farmers in Tanzania as also observed by Tarekegn et al. (2018) for Cameroonian goats. Lack of proper and structured breeding programs with clear breeding strategies led to lack of population genetic structure observed in the present study. The movement of livestock keepers with their animals in search of water and pasture has been one of the main defining characteristics of the pastoral production system in which most of the goat production in Tanzania takes place. Even in the Southern regions of Tanzania particularly Mtwara and Lindi where livestock production was relatively less common have experienced a rapid increase in the number of goats partly due to the recent high influx of pastoralists from other regions of Tanzania who have been forced to look for grazing areas bringing into the regions new goat genotypes (Mwambene et al., 2014).

Significant population sub‐structuring was observed when goat populations from different regions of the African continents were considered in the analysis consistent with what was reported for sub‐Saharan African goat breeds with 14% of inter‐population variation using microsatellites markers (Chenyambuga et al., 2002). This is due to physical distance and low level of interaction between some regions of Africa especially between East Africa and West Africa.

### 4.3 Demographic history

The bimodal pattern of distribution observed for each population indicates that the populations were in equilibrium or stable (Hartl, 2004; Rogers & Harpending, 1992) consistent with the neutrality statistics of Tajima's *D* and *F*’s which were non‐significant. A similar demographic pattern has been observed in Ethiopian (Tarekegn et al., 2018) and Oman indigenous goats (Al‐Araimi et al., 2017), but not in Nigerian goats (Awotunde et al., 2015; Okpeku et al., 2016). On the contrary, the Harpending's r and SSD values supported population expansion. A separate analysis for Haplogroups revealed a smooth unimodal distribution for Haplogroup A indicating population expansion consistent with some previous studies (Hou et al., 2008; Joshi et al., 2004; Zhao et al., 2011) but contrary to other studies (Kibegwa et al., 2015; Tarekegn et al., 2018) which reported unimodal peaks for Haplogroup A. Bimodal peaks though not very distinct, were observed for Haplogroup B and G similar to what was observed previously (ibid) for Ethiopian and Kenyan goats implying a mutation–drift equilibrium for the haplogroups.

## 5 CONCLUSION

This is the first study that investigates the genetic diversity within and between the indigenous goat populations using samples from all the agro‐ecological zones where goat production is practiced in Tanzania. The goats have high genetic diversity and come from three maternal origins A, B, and G with the majority of them originating from Haplogroup A. There is a lot of admixture and very low genetic variation between populations. Population expansion occurred in Haplogroup A and the individual populations. The high within‐population diversity observed for the Tanzanian goats could present an abundant resource for selective breeding in the different agro‐ecological regions of the country when planning and implementing community‐based breeding programs in which farmers select and breed animals within their herds.

Information generated in this study provides a valuable tool for conservation strategies and the data herein indicate that for many of the populations, the inherent genetic diversity has been successfully maintained. The adaptive traits and other unique features in these populations need to be well studied, understood, and preserved in the breed improvement programs as a strategy for conservation of animal genetic resources.

## CONFLICT OF INTEREST

There are no conflicts of interest between authors, and permission from each author has been granted.

## AUTHOR CONTRIBUTIONS

**Athumani Nguluma:** Data curation (lead); Formal analysis (lead); Investigation (equal); Methodology (equal); Writing‐original draft (lead); Writing‐review & editing (lead). **Martina Kyalo:** Data curation (supporting); Formal analysis (supporting); Methodology (supporting); Supervision (equal); Writing‐review & editing (supporting). **Getinet Mekuriaw Tarekegn:** Conceptualization (equal); Formal analysis (supporting); Writing‐review & editing (supporting). **Rose Loina:** Data curation (equal); Formal analysis (equal); Methodology (equal); Writing‐review & editing (supporting). **Zabron Nziku:** Formal analysis (supporting); Writing‐original draft (supporting); Writing‐review & editing (supporting). **Sebastian Chenyambuga:** Formal analysis (supporting); Supervision (supporting); Writing‐review & editing (supporting). **Roger Pelle:** Conceptualization (lead); Data curation (supporting); Formal analysis (supporting); Funding acquisition (lead); Investigation (lead); Methodology (supporting); Project administration (lead); Resources (lead); Supervision (lead); Validation (equal); Writing‐review & editing (supporting).

## Supporting information

Figure S1

Figure S2

Figure S3

## Data Availability

Mitochondrial sequence data generated as part of this project are deposited in dryad and given accession number https://doi.org/10.5061/dryad.w3r2280rn. Other mtDNA sequences incorporated into the analysis were downloaded from this source and can be retrieved as per the relevant citations.
