# Supplementary material for: Mitochondrial DNA D‐loop sequence analysis reveals high variation and multiple maternal origins of indigenous Tanzanian goat populations
Source: Ecol Evol. 2021 Nov 1;11(22):15961–71. doi: 10.1002/ece3.8265 (PMC8601934; doi:10.1002/ece3.8265)
Supplement: Supplementary file 2 — Figure S2 [file ECE3-11-15961-s002.docx]

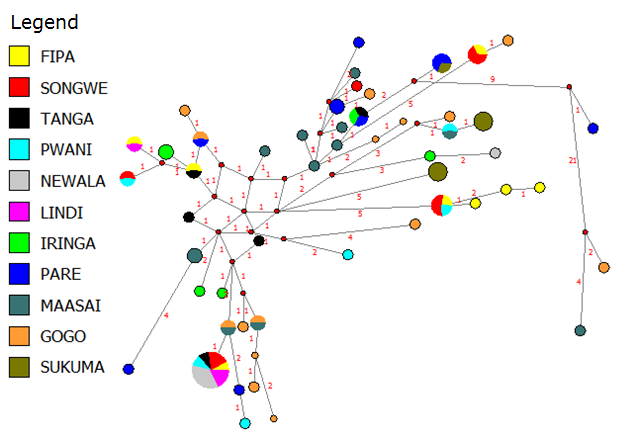
 **Supplementary Figure 2.** Median-joining network analysis for Haplogroup G of indigenous goat populations of Tanzania showing sharing of haplotypes between different goat populations. The area of the circle is proportional to haplotype frequency.
