# Supplementary material for: Mitochondrial DNA D‐loop sequence analysis reveals high variation and multiple maternal origins of indigenous Tanzanian goat populations
Source: Ecol Evol. 2021 Nov 1;11(22):15961–71. doi: 10.1002/ece3.8265 (PMC8601934; doi:10.1002/ece3.8265)
Supplement: Supplementary file 3 — Figure S3 [file ECE3-11-15961-s001.docx]

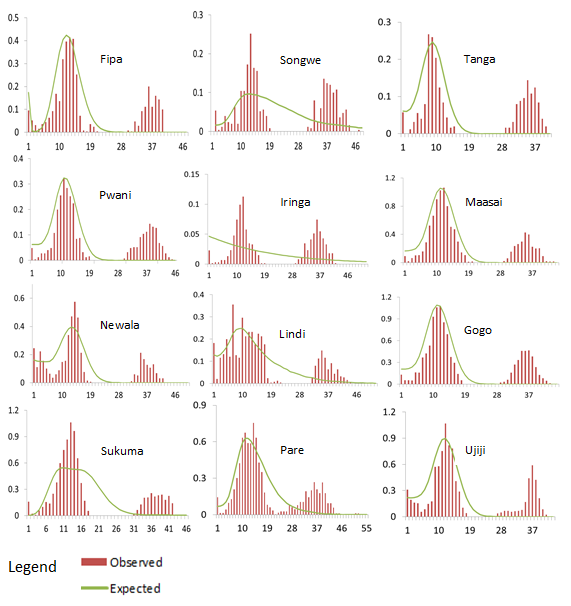
 **Supplementary figure 3.** Mismatch distribution graphs for the 12 Tanzanian indigenous goat populations analyzed in this study. The x axis shows the number of pairwise differences, the y axis shows the frequency of the pairwise comparisons.
